# A case analysis of partnered research on palliative care for refugees in Jordan and Rwanda

**DOI:** 10.1186/s13031-020-00333-6

**Published:** 2021-01-06

**Authors:** Sonya de Laat, Olive Wahoush, Rania Jaber, Wejdan Khater, Emmanuel Musoni, Ibraheem Abu Siam, Lisa Schwartz, the Humanitarian Health Ethics Research Group, Matthew Hunt, Lynda Redwood-Campbell, Laurie Elit, Elysée Nouvet, Rachel Yantzi, Kevin Bezanson, Carrie Bernard, Takhliq Amir, Ani Chénier, Gautham Krishnaraj, Corinne SchusterWallace

**Affiliations:** 1 Global Health, McMaster University, MDCL 3500, 1280 Main Street West, Hamilton, ON L8S 4K1 Canada; 2 School of Nursing, McMaster University, Hamilton, ON Canada; 3 Department of Philosophy, Institute on Ethics & Policy for Innovation, McMaster University, Hamilton, ON Canada; 4 School of Nursing, Jordan University of Science and Technology, Irbid, Jordan; 5 University Teaching Hospital, Kigali, Rwanda; 6 United Nations High Commission for Refugees, Amman, Jordan; 7 Department of Health Research Methods, Evidence and Impact, McMaster University, Hamilton, ON Canada; 8 School of Physical and Occupational Therapy, McGill University, Montréal, QC Canada; 9 Family Medicine, McMaster University, Hamilton, ON Canada; 10 Department of Obstetrics and Gynecology, McMaster University, Hamilton, ON Canada; 11 School of Health Studies, Western University, London, ON Canada; 12 Thunder Bay Regional Health Centre, Thunder Bay, ON Canada; 13 Department of Family and Community Medicine, University of Toronto, Toronto, ON Canada; 14 Geography and Planning, University of Saskatchewan, Saskatoon, SK Canada

**Keywords:** Research collaboration, Ethical partnership, Palliative care, Humanitarian crisis, Ethics, Refugees

## Abstract

**Background:**

This case analysis describes dilemmas and challenges of ethical partnering encountered in the process of conducting a research study that explored moral and practical dimensions of palliative care in humanitarian crisis settings. Two contexts are the focus of this case analysis: Jordan, an acute conflict-induced refugee situation, and Rwanda, a protracted conflict-induced refugee setting. The study’s main goal was to better understand ways humanitarian organizations and health care providers might best support ethically and contextually appropriate palliative care in humanitarian contexts. An unintended outcome of the research was learning lessons about ethical dimensions of transnational research partnerships, which is the focus of this case analysis.

**Discussion:**

There exist ongoing challenges for international collaborative research in humanitarian conflict-induced settings. Research partnerships were crucial for connecting with key stakeholders associated with the full study (e.g., refugees with life limiting illness, local healthcare providers, aid organization representatives). While important relationships were established, obstacles limited our abilities to fully attain the type of mutual partnership we aimed for. Unique challenges faced during the research included: (a) building, nurturing and sustaining respectful and equitable research partnerships between collaborators in contexts of cultural difference and global inequality; (b) appropriate ethics review and challenges of responding to local decision-maker’s research needs; and (c) equity and fairness towards vulnerable populations. Research strategies were adapted and applied to respond to these challenges with a specific focus on (d) research rewards and restitution.

**Conclusions:**

This case analysis sheds light on the importance of understanding cultural norms in all research roles, building relationships with decision makers, and developing teams that include researchers from within humanitarian crisis settings to ensure that mutually beneficial research outcomes are ethical as well as culturally and contextually relevant.

## Background

### Humanitarian context

The topic of this case analysis—dilemmas and challenges of ethical partnering—emerged out of research conducted by the Humanitarian Health Ethics Research Group (HHERG), based in Canada, on the moral and practical dimensions of providing palliative care to refugees residing in camps in Jordan and Rwanda. In humanitarian crises, the priority can be to divert scarce resources to those most likely to survive. While lifesaving ought to remain the priority of healthcare, the unfortunate reality is that within humanitarian contexts some people will not survive due to illness or injury. Recently humanitarian aid organizations and healthcare providers (HCP) have begun to recognize the benefits of palliative care in humanitarian crisis as a means of achieving the humanitarian goal of reducing suffering [1–5]. Palliative care has also been recognized in various global settings for its cost effectiveness as well, particularly among patients with complex comorbidities [6, 7]. Within resource limited humanitarian settings including refugee camps, low- or no-cost palliative care interventions are increasingly being explored for their high impact relative to cost [8–10]. To date, little evidence exists to understand the experiences and needs of HCP, their patients and patient’s families regarding the provision of palliative care in complex crisis settings. Humanitarian aid organizations and HCP require a framework for providing palliative care in humanitarian settings based on such evidence so they can offer something even when it appears, as one HCP we interviewed stated, “there is nothing left to offer.” International partnering was essential for generating contextually relevant findings, but it did not come without its challenges. This case analysis focuses on challenges and benefits of developing partnerships in two humanitarian contexts.

For the purposes of study, Jordan was selected as a site representative of refugees fleeing acute conflict and economic crises in the surrounding area [11, 12]. Jordan has a long history of hosting refugees from conflicts in neighbouring countries (e.g., Palestine, Iraq, Yemen and Sudan) and is one of the main host countries for refugees escaping the war in Syria. The conflict in Syria was considered acute at the time the research project began though now it can be considered protracted as it has persisted since 2011. Jordan was also selected because of prior collaborations with scholars there. One of the authors (OW) lived and worked there previously and had close ties in the country. In addition, palliative care services were increasing for the general population and were available to refugees in some urban settings and in the camps. The Syrian refugee population is one of the largest refugee movements in the world. The influx of forcibly displaced people into Jordan has been significant with more that 650,000 registered refugees and estimates of a total population of more than 1.3 million displaced Syrians, including those who are not registered [13]. This large population movement has resulted in depleted resources, greater job competition, overburdened infrastructure, and strained social services such as healthcare and education, which were already struggling before this refugee crisis [14]. The majority of Syrian refugees (80%) live in the larger cities in the north and the capital Amman with less than 20% living in the refugee camps in the north of the country; the remainder are scattered in other communities in Jordan [13]. At the time of the study, Syrian refugees with life threating illness or serious heath related suffering were able to access healthcare similar to the local population and palliative care through two Jordanian palliative care non-governmental organizations [15]. Those residing outside of the camp were able to receive a voucher for healthcare if they were registered with the UNHCR. The voucher covered costs at the rate of healthcare services for uninsured Jordanians.^1^

Rwanda was selected as a site representative of refugees of protracted conflicts. A generous host to refugees from ongoing and recurrent conflicts for much of the past two decades, there are camps in Rwanda that are nearly 25 years old, while the most recent one was established in 2015 [16–18]. Of the approximately 172,000 refugees in Rwanda, the majority are Congolese or Burundian [19, 20]. Although 90% of refugees in Rwanda reside in camps, there is considerable integration among the host and refugee populations [21]. With the influx of these new inhabitants, there have been strains on local education and health systems, along with accelerated natural resource depletion and erosion particularly in mountainous regions of refugee settlement. These impacts, however, are seen as being offset by economic benefits. Cash aid provided to refugees has had positive spillover effects on the local community, contributing to greater social integration [22–24]. With basic healthcare provided in clinics situated within refugee camps, inhabitants also have access to the same secondary and tertiary care as do Rwandan nationals. The camp clinics, particularly those in remote parts of the country, also offer services to Rwandan nationals living near the camps [23–25]. In terms of palliative care, Rwanda emerged as a leader in Africa in developing a national palliative care strategy in 2011. There is a centralized national plan coordinated through the Rwandan Biomedical Centre and the Ministry of Health to support community-based palliative care along with more comprehensive hospital-based care and greater access to opioid pain relief [26, 27]. Taking a human rights approach to palliative care, it is seen as “restoring the sense of humanity and dignity lost during the genocide against Tutsi, because it is focused on the person and not only on the disease” [26].

### Research study

The case analysis emerged as an unexpected outcome of a program of research reported on elsewhere [11, 28–31]. The main research questions guiding that program of research included: How can humanitarian organizations best support ethically and contextually appropriate palliative care in humanitarian crises? What are the ethical complexities of doing so? How can existing standards be adapted to support delivery of ethically and contextually appropriate palliative care in humanitarian action [11]? The study questions and objectives were developed in dialogue with representatives from international aid agencies, local non-governmental aid organizations, and individual healthcare providers and policy makers associated with palliative care in crisis settings and with refugee healthcare through the following concurrent mixed methods: (a) a critical interpretive synthesis literature review of palliative care needs, interventions and challenges in humanitarian crisis settings [28], (b) a survey of whether humanitarian organizations provide palliative care and the extent they enable their staff to provide it [29], (c) in-depth interviews that enabled exploration of international HCPs moral experiences in situations of extreme suffering [30], and (d) a thematic analysis of the interviews and survey leading to a summary of obstacles to the provision of palliative care in humanitarian crisis contexts [31].

These elements preceded and informed the selection of the four thematically distinct case studies—public health emergencies, natural disasters, acute conflict and protracted conflict—that allowed us to explore the experiences of care for those severely suffering or likely to die in humanitarian settings. Breadth and diversity of experience was prioritized in the design of the research with a goal of identifying findings and recommendations relevant to a variety of refugee and conflict settings. Information was collected through open-ended interviews with humanitarian organization staff (local and international) (*n* = 3), local care providers (*n* = 7), humanitarian policy-makers and managers (*n* = 3) working locally and internationally, along with refugees living with life threatening conditions and their family (*n* = 24) to better understand current norms and lived experiences of palliative care needs in camp and community settings. Findings of the program of research drew attention to context-dependent barriers to the provision of palliative care [28–31].

Members of HHERG are based at Canadian universities (McMaster and McGill). At the outset of the research it was clear that partnerships with research collaborators in the country settings would be the lifeblood of the program of study: a vital means of advancing research on palliative care in refugee settings. While the initial research plans originated in Canada, the intention was never for it to be the locus of decisions-making or direction. Collaboration with researchers in Jordan and Rwanda would be the only way to achieve research of relevance to those residing and working within those—or similar—humanitarian crisis contexts. Along the way, important learning emerged about the challenges of building strong, mutual collaborations across multiple countries, cultures and institutions.

### Scientific importance

Our research was conducted in active humanitarian settings rather than in more stable settings because complex conditions of international humanitarian crises necessitate the development of context-dependent responses from humanitarian service providers. Partnerships and collaborations were essential to inform our research strategies, ensure that study approaches were acceptable and ethically sound, complete the study, and to understand the context, needs and opportunities for service providers and the populations they serve [32, 33]. With more than 79 million people displaced from their home contexts, humanitarian response needs to be attentive to severe suffering and to respond appropriately by providing care that is sensitive to the culture and the unique needs of the displaced [34]. The range of causes of life-limiting and life-threatening conditions include violence and injury, malnutrition, infection and illness, along with non-communicable diseases [35]. Palliative care in humanitarian settings holds promise of supporting overwhelmed health systems by addressing physical, psychosocial and spiritual suffering through a variety of means, including ones of low cost and high impact [1–4, 10]. For instance, our research uncovered that patient accompaniment (e.g., sitting by the bedside) or childcare for a sick parent were small acts that could greatly support palliative patients and their families [11]. These small things, however, do not diminish the importance larger system-wide improvements that can reduce need. In line with sentiments expressed by the Lancet Commission on Palliative Care and Pain Relief [1], we also recognize that along with ‘small things,’ global economic disparities and regulatory frameworks rooted in historical and ongoing patterns of discrimination and bias need to be addressed. In building the equitable partnership there is greater chance that palliative care findings and interventions stemming from the research will have more relevance and be more sustainable within affected communities. Already, findings from the research have informed the World Health Organization guideline development and updates to the Sphere humanitarian standards [5, 36] (e.g., Sphere Minimum Standards, section 2.7 of the Essential Concepts in Health). They have also been shared among the partners and collaborators and their respective organizations.

### Challenges to research

#### Building, nurturing and sustaining respectful and equitable research partnerships

Admittedly, the research team emerged in a lopsided manner, with the bulk of early decision-making residing with the Canadian team members. This, however, is a natural reality in the establishment of collaborations: partnership do not emerge fully formed. They require initiation, nurturance and growth. In developing the research partnerships for this study and building the collaboration, emerging partners had an opportunity to review and discuss tools such the R2HC research ethics tool and the Canadian Coalition for Global Health Research partnership assessment tool and principles of global health research [32, 33, 37]. These resources proved helpful in preparing collaborators on issues of importance to discuss at the outset and throughout the development of the research partnership. An early challenge was that the research topic did not resonate with each of the individuals approached to become collaborators. In particular, the topic of palliative care did not appear as relevant to those who were managing and mitigating multiples issues of importance to refugee care (e.g., security, food, shelter). As one collaborator explained, “[the] first time [I was] approached [I] felt that the area of interest should not be at focus of humanitarian actors who have more life threatening situations, needs to be addressed, but later when the findings came up I felt it’s [a] neglected area by most stakeholders especially with protracted crisis that’s been extended for more than two years.” Although not all partners saw the research topic as valid, there was trust and respect among host-country partners, enabling the research to proceed. Fortunately, as it unfolded, continued discussion of findings with collaborators resulted in full consensus on the value of the research. That experience, however, raises the question: would we have been perpetuating historical wrongs had the sentiments expressed by that one collaborator been repeated by others, or had they not changed their view when findings became available?

From the outset, the research team was committed to grappling with power differentials, risks of unconscious biases, and legacies of colonial assumptions. Despite our attention to the locus of power in developing the partnership, there remained cultural, historical and contextual factors that presented the team with underlying challenges. Mechanisms of inequality including longstanding political processes and social practices that set racial hierarchies and economic barriers for countries, specific cultures or groups, and for individuals. The legacies and impact of such processes presented the research team with perceived and actual degrees of distrust or potential concerns of exploitation. Making a space to discuss uncomfortable realities led to difficult but fruitful discussions that had bearing on research design, intellectual property, financial compensation, and division of labour [38]. In sharing the lived experiences of working toward a more just and equitable ethical partnership, the aim is to support ongoing and future endeavours meant to overcome mechanisms of global inequities that continue to set partners apart. Meant initially to establish a strong collaboration based in equity and mutual beneficence, the discussions further clarified expectations from the host researchers and identified local institution or agency priorities.

#### Ethics review and challenges of responding to local decision-maker’s research needs

Initial ethics approval was granted by the two Canadian research intuitions affiliated with the project, with the understanding that amendments would be submitted for each subsequent country brought into the study. Amendments in the form of translated consent forms and modified recruitment procedures were anticipated. As a research lead in Jordan said, “Although we have a clear interview guide and protocol in this research, some aspects needed to be modified to fit our culture*.*” Beyond recruitment and consent processes, the study was further modified to reflect locally identified needs and sensitivities. For instance, the study design in Jordan was adjusted based on a request from the Ministry of the Interior who requested an expansion to include interviews with Jordanians about their palliative care experiences. This was to minimize the risk of perceptions that refugees were getting preferential treatment and it had the added benefit of providing comparative data with which to analyze refugees’ experiences. Fortunately, we were able to accommodate the request as it did not add greatly to the research budget.

In both Jordan and Rwanda, the ethics approval processes required cultural navigators to identify required levels of approvals, and the order in which to obtain them. Collaboration with team members in the country settings ensured no steps were missed that could otherwise delay or derail the research. In Jordan, ethics reviews were also completed by the Ministry of Health, the Jordan University of Science and Technology and UNHCR, the sequencing of these approvals was informed by our research partner and UNHCR lead. Permission was also required for each researcher to be able to enter the camp to complete interviews and was obtained with support from our collaborating partners at UNHCR. In Rwanda, ethics review and approvals were obtained from the Rwandan National Ethics Board, the Ministry of Emergency and Refugee Affairs (MIDIMAR, now MINEMA), the Ministry of Health and the aid organizations providing healthcare in the camps.

#### Equity and fairness towards vulnerable populations

To support our aim of equity and fairness towards vulnerable populations, numerous team discussions were had—and revisited—to determine appropriate means of responding to challenges related to cultural norms of talking about serious health issues and around fair participant compensations. In both country settings, it is considered a cultural taboo to talk about serious illness, dying and death. It was also a challenge to interview refugee participants who suffer compounded vulnerabilities of displacement and terminal illness, and who had little to no idea about the concept of palliative care. According to one collaborator, “The main challenge in doing a research in palliative care in Jordan is the lack of knowledge in palliative care so we had to explain what palliative care is before starting our interviews*.*” Explaining palliative care to patients or their family members had to be done with cultural sensitivity, and—more importantly—so as not to inadvertently disclose a person’s health status. In this regard, the interview guide had to be modified to reflect culturally appropriate language, focusing on norms, expectations or experiences around healthcare. Nevertheless, excluding people because of their vulnerability was out of the question. To do so would mean losing their perspectives what was pertinent to them. Excluding them would have been unfair and would only reinforce their vulnerability. Extensive discussions and training were done with refugee care professionals, healthcare providers and scholars skilled in crisis-setting research about best-practices and context-dependent approaches to interviewing seriously ill refugees and their families. The priority was to minimize distress for interview participants, avoid (re-)traumatization, health status disclosure, or false hopes whilst also ensuring they had a fair opportunity to be heard. Extra support such as debriefing meetings were needed for interviewers during the data collection phase in Jordan in particular. The research assistants conducting interviews there were nurses experienced with palliative care but not with refugees. They were surprised at how emotionally difficult interviewing was with people needing end of life care and living in humanitarian crisis settings.

In terms of participant compensation, monetary compensation was not permitted for participants in Jordan. While several on the research team considered some form of material compensation as fair, a reciprocal act. The rationale from the authorising body was that this was a means to ensuring research would not become a commodity item. The compensation we were able to provide participants was information about supportive local services. With the Rwanda case, there was much discussion about appropriate compensation to refugee participants. Which was most appropriate: cash or in-kind goods (particularly hard to find items)? Would the compensation negatively impact socio-economic relations among individuals or groups in the camps? After careful deliberation that involved local HCP, camp staff who were familiar with the social setting within the camps and with local and international academics familiar with research in Rwanda, a decision for cash-based compensation was made. It was a decision founded on respecting the dignity and autonomy of the refugee participants, and a means of demonstrating a fair reciprocation for the time and perspective they offered. The amount was determined by our local research partners based on cultural expectations of an amount that was respectful but still well within the range of the (relatively low) wage expectations of the majority of Rwandans*.* Local input in both countries helped us understand rationales for approaches to compensation, and make culturally appropriate offerings, and provide equitable but also safe compensation for participants.

### Research strategies

Research that crosses borders presents additional challenges in managing teams effectively. Along with the challenges presented above, in this study we encountered barriers in each of the six areas Pischke et al. described as having been encountered in their research [39]. Like them, we encountered barriers related to money transfers (timeliness of transfers and currency conversion rates), bureaucracy (visas, permits to enter camps and ethics reviews), safety (research assistants in the field)), differing cultural traditions (awareness of anticipated illness outcomes, working days, gender norms), language, and fieldwork logistics (transportation). These challenges were managed through collaborative discussions and in following the advice from partners situated in each setting. Discussions also included important recognitions and acknowledgements such as authorship on publications, and other dissemination activities so that expectations could be clarified early.

Despite the lopsided beginnings of the research collaborations, the team resolved to communicate regularly, through a variety of means and with transparency to overcome the initial power imbalance and to address any underlying potential or actual cultural, historical and contextual challenges. The nature of the refugee camp context in which the research was set attuned us to vulnerabilities, power differentials and risks of unconscious biases or legacies of colonial assumptions impacting our practices and overall partnership. Transparency with respect to funding and operations along with an ability to share final findings with local health practitioners and government ministries created a space to overcome historic power imbalances. Team members checked in regularly as a group to discuss study progress and to address any actual or perceived issues such as cultural translation or power relations to foster a collaborative environment among the full research team. Unsatisfied with the perpetuation of hierarchies through language such as “global north” and “global south”, team members engaged in continuous, open dialogue about expectations of being in the partnership, anxieties and concerns around a sense of colonial/empirical imposition, and acknowledgement of global and historic inequities (at structural and systemic levels).

Whereas collaborators who joined in the project later in the process described feeling disconnected from the team, the more they were invited to share ideas, thoughts and skill in the evolving project, the more feeling of full membership emerged. Indeed, one member recalls his earlier sense of feeling removed as creating confusion for himself. He recognized his perception had to change, that “I should start feeling the ownership of the project too,” resulting in more active and equitable participation. Frustratingly, even when feelings of authentic partnering were strengthening, federally regulated travel visas were denied to collaborators, preventing them from being able to participate in in-person team meetings or knowledge transfer activities in Europe and North America. Thus, reinforcing the tragic reality of persistent global inequities, despite the teams attempts to overcome them.

Partners in Canada, who initiated the research, helped ease the burden of tasks collaborators at the research sites were best situated to complete (e.g., REB submissions) by preparing presentations and drafting authorization-request letters from afar. Thus, any sense of imposition was diminished as collaborators maintained open dialogue about concerns and about the mutual benefits of the collaboration, resulting in one case-study lead saying that the work sharing was “not a burden, but [a] light load.”

In terms of finances, the unintended feeling of restricting the independence of collaborators by managing funds in Canada was minimized, to the extent possible, through open discussions of budgets. Expenses incurred by partners at a geographical distance from the finance-managing institution were also not micromanaged but responsive to local practices, for example, in Jordan, paying the research assistants on a bi-weekly basis as opposed to waiting till then end of the data collection phase. The regular contact aided collaborators maintain a sense of being valued members of the team.

#### Research rewards & restitution

In research, it is predominantly the findings or their uptake in policy or practice that is considered the reward. In the case of this partnership, the reward was the outcome of greater international collaboration possibilities. Failure to recognize host-country researchers or only including their names among the author list in final publications, has been cited as signs of unethical transcontinental and cross-cultural research practices [40–43]. At all stages of the research and dissemination, team members from all sites were involved in adapting the research design to specific contexts, in analysis, and in acknowledgment and recognition in publications and presentations. Likewise, team members from Canada, Jordan and Rwanda were integrally involved in analysis, presentations, conferences, and publications. As one of the partners explained, “When I was invited to collaborate in this research, I was excited since this is my area of interest and an area that is needed to be assessed... For me this research is the first one that I have an experience to work in collaboration with other researchers from other countries... Fortunately, this research opens a new opportunity for me where I am now working in a project with researchers from [other institutions in other countries].”

All the frustrations that came along with authorizations and approvals, and with the difficulties of navigating research between a team separated by vast geographic distance, the ability to finally meet with refugees and hear their stories made the challenges encountered worthwhile. The ability to connect with and collect information on the experiences of being a seriously ill refugee put into focus the reason why all of the collective work was done. As such, it was vitally important that the findings were shared through traditional academic means (e.g., conference presentations, peer-review publications). Findings were also returned to knowledge-users and decision-makers at each of the site locations in Jordan and Rwanda. Knowledge users and policymakers at all levels—from refugee camp clinic healthcare providers to top-level government and international organization representatives—were kept informed on the findings throughout the research study with the understanding that important information critical to improving care for seriously ill refugees needs sharing in a timely way [11].

## Conclusion

In the best interest of those suffering or likely to die in conflict-induced humanitarian crisis settings, international collaborators should support local needs and engage in partnerships and collaborations, adapting research protocols as needed. As this case analysis describes, involvement of local research collaborators was essential for everything from research design and contextualizing findings, to practical processes such as hiring and training team members and for accessing the population the study was meant to serve. Common experiences across the study settings included the importance of building relations that are sensitive to multiple vulnerabilities and unequal power among research partners as much as with research participants, and the need to create spaces that facilitate the sharing of knowledge and experiences. The main outcomes of this case analysis include: the importance of understanding cultural norms in all research roles; considerations of how to promote the integrity and ethics of the research; and the importance of training, reflection and discussion to support research team members and to promote participant voices in research with refugees. The importance of supporting team members during the research process was critical for ensuring quality data, grounded interpretation of participant information and translating the understanding of the results for culturally sensitive dissemination. The need for ongoing communication between international collaborators is vital for all future partnerships. Despite global social and political forces with longstanding roots that continue to hold barriers in place, active and engaged participation towards the goal of equitable partnering has been our attempt to overcome mechanisms of global inequities in order to bring partners together.

## Data Availability

De-identified transcript data will be made available when the project is officially completed including overall analysis of all data and publication on the McMaster dataverse portal (http://dataverse.scholarsportal.info/dvn/).

## Abbreviations

HCPs: Healthcare providers
SES: Socioeconomic status
WHO: World Health Organization
UNHCR: United Nations High Commissioner for Refugees
PC: Palliative Care
MOH: Ministry of Health
MSF: Doctors without borders
REB: Research Ethics Board

## Footnotes

1 Shortly after the completion of data collection, the Jordanian government’s policy changed, requiring Syrian refugees to pay the foreign rate for access to health services outside of the camps. This policy has since been reversed with restoration of access to health services at the uninsured Jordanian rate since April 2019 (Dec 31, 2019 https://www.unhcr.org/jo/12449-unhcr-continues-to-support-refugees-in-jordan-throughout-2019.html )
